# Eliciting and Recording Event Related Potentials (ERPs) in Behaviourally Unresponsive Populations: A Retrospective Commentary on Critical Factors

**DOI:** 10.3390/brainsci11070835

**Published:** 2021-06-24

**Authors:** Alexander Rokos, Richard Mah, Rober Boshra, Amabilis Harrison, Tsee Leng Choy, Stefanie Blain-Moraes, John F. Connolly

**Affiliations:** 1 Integrated Program in Neuroscience, McGill University, Montreal, QC H3A 2B4, Canada; alexander.rokos@mail.mcgill.ca; 2 Department of Linguistics and Languages, McMaster University, Hamilton, ON L8S 4M2, Canada; mahrl@mcmaster.ca; 3 School of Biomedical Engineering, McMaster University, Hamilton, ON L8S 4L8, Canada; boshrar@mcmaster.ca; 4 Imaging Research Centre, St. Joseph’s Healthcare Hamilton, Hamilton, ON L8N 4A6, Canada; amabilis.harrison@gmail.com; 5 McMaster Integrative Neuroscience Discovery & Study (MiNDS), McMaster University, Hamilton, ON L8S 4L8, Canada; tlchoy@hotmail.com; 6 School of Physical and Occupational Therapy, McGill University, Montreal, QC H3G 1Y5, Canada; 7 Centre for Advanced Research in Experimental and Applied Linguistics, Department of Psychology, Neuroscience & Behaviour, Hamilton, ON L8S 4K1, Canada; jfcnep@gmail.com

**Keywords:** disorders of consciousness, electroencephalography, N400, P3b, event related potentials

## Abstract

A consistent limitation when designing event-related potential paradigms and interpreting results is a lack of consideration of the multivariate factors that affect their elicitation and detection in behaviorally unresponsive individuals. This paper provides a retrospective commentary on three factors that influence the presence and morphology of long-latency event-related potentials—the P3b and N400. We analyze event-related potentials derived from electroencephalographic (EEG) data collected from small groups of healthy youth and healthy elderly to illustrate the effect of paradigm strength and subject age; we analyze ERPs collected from an individual with severe traumatic brain injury to illustrate the effect of stimulus presentation speed. Based on these critical factors, we support that: (1) the strongest paradigms should be used to elicit event-related potentials in unresponsive populations; (2) interpretation of event-related potential results should account for participant age; and (3) speed of stimulus presentation should be slower in unresponsive individuals. The application of these practices when eliciting and recording event-related potentials in unresponsive individuals will help to minimize result interpretation ambiguity, increase confidence in conclusions, and advance the understanding of the relationship between long-latency event-related potentials and states of consciousness.

## 1. Introduction

In clinical practice, patient unresponsiveness is typically attributed to unconsciousness. However, an individual’s ability to respond may be attenuated by many factors, including motor injury, sedation, pain, tiredness, and delirium. As a result, upwards of 40% of unresponsive patients are incorrectly diagnosed as unconscious [1,2,3,4]. These unresponsive individuals in fact have varying levels of conscious awareness of their environment, ranging from none (e.g., unresponsive wakefulness syndrome/vegetative state (UWS/VS)), to liminal (e.g., minimal conscious state (MCS)) to full (e.g., locked in syndrome (LIS)) [5,6,7]. Misdiagnosis of these disorders of consciousness (DOC) have grave consequences, including hastily removing individuals with potential for recovery from life support, or putting them into nursing home-like facilities without being given the proper opportunity for rehabilitation [8].

Recent progress has been made using neuroimaging techniques to categorize an individual’s consciousness directly from their brain activity, thus bypassing the need for a behavioral response [9,10,11]. The brain activity of an unresponsive individual can be recorded through a variety of techniques, most commonly functional magnetic resonance imaging (fMRI) and electroencephalography (EEG) [12]. These techniques have been used to demonstrate that a handful of individuals who were putatively unconscious (i.e., diagnosed with unresponsive wakefulness syndrome) have the ability to follow commands [13,14]. The discovery of neurophysiological evidence of conscious awareness in the absence of behavioural responsiveness has perturbed the current taxonomies of disorders of consciousness, and prompted calls to include brain-based evidence in diagnostic and assessment standards [15,16]. While such calls have yet to be implemented in clinical practice, it has become increasingly important to refine the processes involved in the elicitation and analysis of brain-based measures of consciousness. In particular, event-related potentials (ERPs) have been used as non-behavioral markers of both states and contents of consciousness. The state of consciousness, which is determined by an individual’s level of wakefulness and awareness, varies from coma and slow-wave sleep to full awareness of the environment, and is linked to the presence or absence of a subjective experience. In contrast, the contents of consciousness refers to the information to which an individual has consciousness access and/or has consciously processed (e.g., “I was not conscious of the red light”) [17]. The work presented in this paper focuses on the relationship between ERPs and an individual’s state of consciousness. Reflecting on previously collected data, we provide an evidence-supported commentary on factors that influence the presence and morphology of two prominent long-latency ERPs, the P300 and the N400, in behaviourally unresponsive individuals. When recording from this population, accurate interpretations of states of consciousness are critically dependent on taking these factors into account during the elicitation and interpretation of ERPs.

Event related potentials are often grouped depending on their latency following stimulus onset. Early ERP components (such as the N1, P2, and mismatch negativity) occur less than 250 ms post stimuli. On the other hand, long-latency components such as the P300 and N400 occur over 250 ms post stimuli. Different ERP components are elicited in response to different cognitive processes [18]. The N1 and P2 are elicited by the auditory system’s response to the physical characteristics of a sound [19]. The N1 is visualized as a negative deflection from pre-stimulus baseline EEG approximately 100 ms following stimulus onset whereas the P2 is visualized by a positive deflection approximately 200 ms following stimulus onset. The N1/P2 response can be elicited by almost any auditory stimuli [20] and may be used to confirm that the stimuli are being received and processed by the auditory system. The mismatch negativity (MMN) is elicited, independent of an individual’s active attention, in response to a mismatching stimulus within a train of identical stimuli (known as an oddball sequence) [21,22,23]. The difference in the EEG response between deviant and standard stimuli yields a negative peak 100–200 ms after stimulus onset, predominantly over frontocentral brain regions [24]. The classic P300 can also be elicited using an oddball sequence and occurs following the onset of an infrequent and task-relevant stimulus [25]. The waveform peaks approximately 250–400 ms post stimulus onset and is most prominent over parietal brain/scalp regions. The P300 consists of two independent components: the P3a, an earlier (c. 250 ms) frontal component reflecting the automatic attraction of attention or an orienting response produced by an unexpected stimulus [26]; and the P3b (i.e., the classic P300), a later (c. 350–400 ms) parietal component, reflecting cognitive workload and the process of context updating, wherein the current stimulus is compared in working memory to the most frequently occurring stimulus in a sequence [27,28]. The N400 waveform is an index of the difficulty of retrieving stored conceptual knowledge associated with a word [29]. It reaches its peak amplitude approximately 400 ms after stimulus onset at midline central or parietal sites [30]. Although interpretations of the N400 vary, the response reflects higher level processing involving attention and language comprehension skills, both of which require conscious awareness. 

Current evidence suggests that there are different brain network dynamics involved in the generation of early and late ERPs. Early cognitively-related ERP components (i.e., <250 ms post-stimuli—the MMN) reflect a perturbation of neural dynamics by sensory input feedforward processing, while late ERP components (i.e., >250 ms post-stimuli—P300 and N400) are mediated by recurrent feedback dynamics in frontoparietal cortices [31,32]. The recurrent feedback pathways which mediate these long-latency ERPs have been reliably associated with the presence of conscious awareness [33,34]. Indeed, it has been shown that early-component ERPs do not reliably differentiate between various levels of consciousness [35,36], while long-latency ERPs provide evidence for the presence of consciousness in unresponsive individuals [9,37,38,39,40,41].

Recent studies have blurred the relationship between the latency of the ERP and the presence/absence of consciousness. Since the P3b is a putative marker of conscious awareness, it theoretically should distinguish between VS/UWS and MCS: some studies have supported this hypothesis [42,43,44], while others did not [35,36,45,46]. The studies yielding contradictory results have been varied. Tzovara et al. reported that global violation ERPs (which are equivalent to the P3b [37]) can be elicited in some comatose patients (i.e., even in the absence of consciousness), yet only in 36% (4/11) of conscious controls [47]. Other studies have demonstrated that the P3b can be elicited even under rigorously subliminal conditions [48], though these results have been contested [49]. The N400 is also a putative marker of conscious awareness, yet Rohaut et al. demonstrated that the N400 was not easily observed on individual conscious controls in a word-word priming paradigm [41]; although, a reliable N400 response baseline in the normative sample was not first established, leading to the peculiar finding of greater N400 activity in the patient sample than in the healthy controls. While multiple explanations for these conflicting results have been proposed, including differences in stimulation paradigms [50,51], and discrepancies of the statistical methods used to analyze electrophysiological signals [49,52], the utility of long-latency ERPs to classify states of consciousness has become unclear. 

Variability in overall ERP paradigm strength [53,54,55] is an ongoing area of interest and controversy in the field. It is clear that there is much to consider when designing effective paradigms for eliciting ERPs in all populations. Some studies outline factors such as stimulus type and frequency which must be taken into account when administering an ERP paradigm [56,57]. Other works have demonstrated that changes in age affect the robustness and morphology of long latency ERPs [53,54,55]. Furthermore, recent studies have tested both active and passive conditions and provide evidence that providing instructions to the participants can influence ERP robustness [58,59]. It is well established that many factors have the ability to influence the effectiveness of any given ERP paradigm. With the current uncertainty surrounding the utility of long-latency ERPs as a means for classifying states of consciousness, consideration of these factors is particularly critical when testing behaviourally unresponsive populations both on an individual, and small sample level. 

In this paper, we provide a retrospective commentary outlining three factors that have influenced the presence and morphology of the P300 and N400 waveforms in our previously collected data: paradigm strength, participant age, and speed of stimulus presentation. These factors are illustrated in three data driven modules. Modules 1 and 2 consist of multiple paradigms presented to small groups of healthy youth and healthy elderly, respectively, and illustrate the effect of paradigm strength and subject age on the presence and morphology of long-latency ERPs. Module 3 is a single-subject analysis of an individual with a severe traumatic brain injury and illustrates the effect of stimulus presentation speed on the presence of the N400. Cumulatively, the presented data represent factors that must be accounted for during the elicitation and interpretation of ERPs in unresponsive populations. We demonstrate that when appropriate steps are taken to maximize the strength of the experimental protocol, long-latency ERPs can be consistently detected in individuals with conscious awareness, even within a single individual or small sample size. As ERPs continue to be used as a tool to probe the state of consciousness of unresponsive individuals, we reiterate that it is critical that the identified factors be taken into account to minimize the ambiguity in result interpretation, and to maximize confidence in conclusions drawn.

## 2. Data Modules 1 & 2: Methods and Results

Previous work presented in Mah & Connolly 2018 [60] validated and assessed the context strength of various commonly used ERP paradigms. Paradigms with stronger contexts (e.g., target words presented within a sentence) were compared to those with weaker contexts (e.g., target words presented in comparison to a single other word). This study was conducted with young and older adults who were recruited from university and senior group environments. Participants were included in the study if they had no previous brain injury and normal or corrected-to-normal hearing. Across large sample sizes, the study demonstrated that paradigms with stronger context features provided better results compared to those with weaker context. Additionally, it was shown that older adults typically present more attenuated ERP responses when compared to younger adults. A randomly selected subpopulation from Mah & Connolly was reassessed in data modules 1 and 2 below in order to assess the validity of these results within smaller, and therefore more clinically relevant, sample sizes.

### 2.1. Module 1: Multiple Paradigms, Healthy Youth

#### 2.1.1. Module 1: Methods 

The effect of the stimulus paradigm on long-latency ERPs can be observed in a randomly selected subpopulation of a group of youth participants (see Mah & Connolly 2018 [60]). Reassessing this small-sample subpopulation, we contrasted two popular methods of eliciting the P300: (1) Modified Fischer (MF) paradigm and (2) Subject’s Own Name (SON) paradigm; as well as two popular methods of eliciting the N400: (1) Semantic Violation Sentences (SVS) paradigm and (2) Word-Word Priming (WWP) paradigm. Participants (n = 5; mean age = 20.2 ± 0.8, range = 18–21; females = 5) were native English speakers with no history of neurological disorders and were dextral.

64-channel EEG was recorded from the participants while they listened to each of the following paradigms:

Modified Fischer paradigm (MF): Participants were presented with a series of pseudorandom auditory stimuli: 80% standard tones (75 ms), 14% deviant (duration difference) (30 ms), 3% familiar novel sound (participant’s own name), and 3% unfamiliar novel sound (dog bark). The standard and deviant tones had a rise/fall time of 5 ms. This paradigm was modified from (Fischer, Dailler, & Morlet, 2008 [61]). 

Names paradigm (SON): Participants listened to a pseudorandom series of 16 words and 1 environmental sound: the subject’s own name, 5 other common first names (two same gender, three opposite gender), an unfamiliar novel sound (dog bark), and 10 high frequency monosyllabic or disyllabic non-salient words of matched length to the subject’s first name. A total of 480 trials were presented to each participant; subject’s own name, other names, and the dog bark were presented 60 times each, and the 10 non-salient other words were presented 6 times each. This paradigm was modified from (Holeckova, Fischer, Giard, Delpuech, & Morlet, 2006 [62]).

Semantic Violation Sentences (SVS): Participants listened to 72 sentences constructed from six to twelve words. The terminal words of each sentence were either semantically congruent or semantically incongruent (e.g., “The pizza was too hot to sing”). This paradigm was adapted from (Connolly & Phillips, 1994 [63]). 

Word-Word Priming (WWP): Participants listened to 160 pairs of words that were either semantically congruent, semantically incongruent, English pseudowords, or reversed English words recorded as “speech noise.” Each pair of words was presented with 1150 ms between words and 3s between trials. This paradigm was adapted from (Holcomb & Neville, 1990 [64]).

During all four paradigms, participants were told that there was no need to pay attention to the stimuli. For participants included in modules 1 and 2: undergraduate participants received course credit and older adults received $20.00 CAD for their participation. All participants provided written informed consent prior to being included in the study. Data collection for all Modules and case studies was approved by the Hamilton Integrated Research Ethics Board who approved the study in accordance with the ethical standards of the Declaration of Helsinki. Complete details pertaining to data collection and each paradigm are described in Mah & Connolly, 2018 [60].

#### 2.1.2. Module 1: Analysis

All recordings were pre-processed using Brain Vision Analyzer 2 (Brain Products GmbH, 2014). Raw data was visually inspected for artifacts and artifact containing epochs were removed. All recordings underwent offline filtering (bandpass 0.1–30 Hz) and ocular artifact correction (via Brain Vision Analyzer 2 *Ocular ICA* transformation).

Trials were segmented by stimulus type. P300 trials (MF and SON paradigms) were segmented into 1200 ms windows (200 ms pre-stimulus to 1000 ms post-stimulus) and N400 trials (SVS and WWP paradigms) were segmented into 1100 ms windows (100 ms pre-stimulus to 1000 ms post-stimulus). All segments were baseline-corrected, and artifacts were automatically rejected by targeting channels with low activity (<0.5 µV), large voltage steps (>50 µV), and large voltage differences (>200 µV within 200 ms) [18]. For each participant, segments were averaged across stimulus type and peak detection was performed on each individual channel (via Brain Vision Analyzer 2’s *Peak Detection* transformation). 

For each paradigm, stimulus type averages were compared on a point-by-point basis using serial t-scores to identify significant differences over time [45,65]. P300 analysis was performed on the difference waves (MF paradigm: subject’s own name—standard tone and bark—standard tone; SON paradigm: subject’s own name—other names, bark—other names, and non-salient other words—other names). N400 analysis was performed on the difference waves between congruent and incongruent sentences for both the SVS and WWP paradigms. Waveform comparisons were conducted using the serial t-score method with differences significant at *p* < 0.05. 

#### 2.1.3. Module 1: Results

P300s induced by the SON paradigm stimulus had a significantly smaller amplitude than those induced by the MF paradigm (MF: 10.6 ± 3.8 µV; SON: 3.3 ± 4.1 µV; *p* = 2.3 × 10^−9^). Both paradigms produced P300s that were not significantly different in latency (MF: 369.7 ± 13.3 ms; SON: 377.9 ± 28.9 ms; *p* = 0.16). P300s induced by the dog bark stimulus had significantly larger amplitude in the MF paradigm than the SON paradigm (MF: 8.8 ± 3.5 µV; SON: 1.0 ± 1.4 µV; *p* = 1.5 × 10^−12^) and a significantly shorter latency (MF: 353.58 ± 59.19 ms; SON: 466.8 ± 51.2 ms; *p* = 1.7 × 10^−5^)

**Figure 1:**
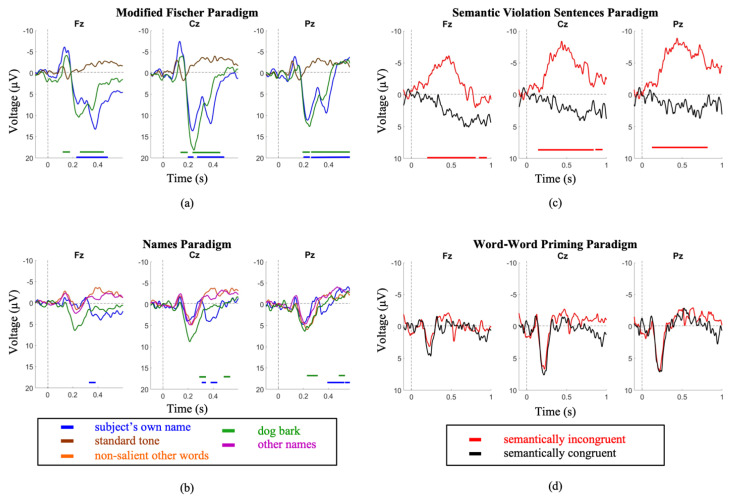
Youth group grand average waveforms. (**a**) A large P300 is elicited by the subject’s own name in the MF paradigm. (**b**) a less robust P300 is elicited by the same stimulus in the SON paradigm. (**c**) A clear N400 is elicited in the SVS paradigm. (**d**) no distinction between conditions is seen in the WWP paradigm. Intervals of significance are indicated by color-matched bars, where the waveform is significant in relation to the standard tone stimulus (MF paradigm), other names stimulus (SON paradigm), and semantically congruent stimulus (SVS paradigm).

Using the serial t-score method to conduct waveform comparisons, it was observed that the SVS paradigm produced significantly distinct waveforms between congruent and incongruent stimulus types (*p* < 0.05), whereas the WWP paradigm did not (Figure 1) 

### 2.2. Module 2: Multiple Paradigms, Healthy Elderly

#### 2.2.1. Module 2: Methods 

Using a second, randomly selected small-sample subpopulation of participants from Mah & Connolly, 2018 [60], we investigated the effect of age on the P300 and N400 responses elicited by the previously outlined paradigms. Five (n = 5) healthy participants over the age of 65 (mean age: 69.8 ± 2.0, range = 68–72; females = 3) listened to all four paradigms while continuous EEG was recorded. Results were compared to the younger cohort (described in Module 1). Written informed consent was obtained from each elderly participant prior to carrying out the experiment.

#### 2.2.2. Module 2: Analysis 

Peak detection as well as within-group paradigm and stimulus-type comparisons were carried out following the same methods described in Module 1 Analysis. 

Waveforms were segmented in 50 ms windows around each peak for amplitude and latency analysis; differences between youth and elderly groups were compared using a repeated measures analysis of variance (ANOVA) as an omnibus test, using group and stimulus type as factors. Greenhouse-Geisser corrections were applied to the degrees of freedom.

#### 2.2.3. Module 2: Results

**Figure 2:**
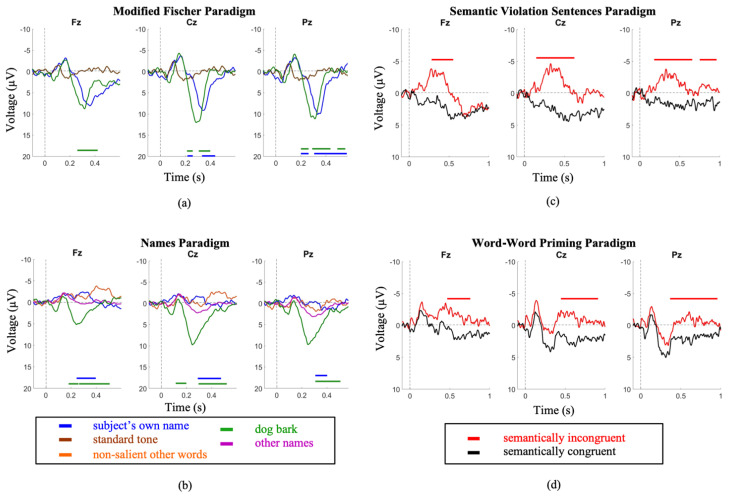
Elderly group grand average waveforms. Intervals of significance indicate where the waveform is significant in relation to (**a**) the standard tone stimulus (MF Paradigm), (**b**) the other names stimulus (SON Paradigm), (**c**) the semantically congruent stimulus (SVS paradigm), and (**d**) the semantically congruent stimulus (WWP paradigm). Time periods where significant differences occurred are indicated by color-matched bars.

Older participants demonstrated similar trends as the younger cohort across both P300 and N400 paradigms. The P300 induced by the subject’s name was significantly larger in amplitude and shorter in latency in the MF paradigm compared to the SON paradigm (Amplitude—MF: 9.3 ± 3.9 µV; SON: 1.9 ± 2.0 µV; *p* = 8.8 × 10^−12^. Latency—MF: 321.3 ± 32.4 ms; SON: 410.8 ± 70.0 ms; *p* = 0.00026). The P300s induced by the dog bark were similarly larger in amplitude and shorter in latency in the MF paradigm (Amplitude—MF: 10.9 ± 2.5 µV SON: 1.7 ± 1.4 µV; *p* = 4.28 x 10^−15^; Latency—Modified Fischer: 321.3 ± 32.4 ms SON: 434.2± 104.6 ms; *p* = 1.1 × 10^−5^) (Figure 2). 

The SVS paradigm elicited a larger amplitude and shorter latency N400 in comparison to the WWP paradigm (Amplitude—SVS: −3.9 ± 3µV WWP: −1.9 ± 2.8µV; *p =* 8.7 × 10^−12^; Latency—SVS: 409.7 ± 82.7 ms WWP: 445.63 ± 32.3 ms; *p* = 0.034))(Figure 2).

**Figure 3:**
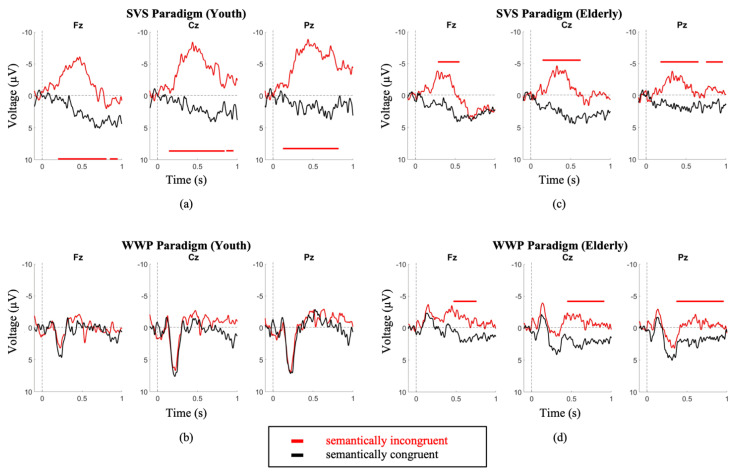
Youth (**a**,**b**) and Elderly (**c**,**d**) group grand average waveforms. the SVS paradigm elicited a larger N400 amplitude in the youth (Youth = −8.7 ± 4 (**a**); Elderly = −3.9 ± 2.8 (**c**); *p* = 3.3 × 10^−2^). The WWP paradigm elicited an N400 in the elderly (**d**) (*p* < 0.05) but not in the youth (**b**). Intervals of significance indicate where the semantically incongruent waveform is significant in relation to the semantically congruent stimulus. Time periods where significant differences occurred are indicated by color-matched bars.

Comparing between youth and elderly groups, the SVS paradigm elicited a larger N400 amplitude in the youth (Elderly = −3.9 ± 2.8; Youth = −8.7 ± 4; *p* = 3.3 × 10^−2^), while the WWP paradigm elicited an N400 in the elderly (*p* < 0.05) but not in the youth (Figure 3).

## 3. Data Module 3: Single Patient with Severe Traumatic Brain Injury, Methods and Results

### 3.1. Module 3: Methods

The effect of speed of stimulus presentation on the N400 ERP can be observed in a case study of a patient with severe traumatic brain injury. The participant was a 46-year-old male involved in a motor vehicle-bicycle collision two months prior to data collection. Computed tomography scans revealed diffuse axonal injury and brain stem hemorrhaging but no epidural or subdural hemorrhage. The patient exhibited shear injury in the left corpus callosum and left superior cerebellar peduncle as well as right sulci subarachnoid hemorrhaging. There was also evidence of bilateral intraventricular hemorrhaging in the occipital horn of the lateral ventricles and a focal hemorrhage in the pons but no uncal herniation or midline shift was present. Due to a pelvic wound, the patient experienced prolonged complications in the hospital including sepsis and urinary tract infections. The patient was sedated in the intensive care unit. Baseline EEG revealed no signs of epileptic seizures.

Data collection for Module 3 was approved by the Hamilton Integrated Research Ethics Board who approved the study in accordance with the ethical standards of the Declaration of Helsinki. Through behavioural testing conducted by physicians and trained medical staff that indicated no signs of consistent yes/no responses or command following, the participant included in Module 3 was determined to be unable to provide informed consent for participation in the study. Written informed consent was obtained through the participant’s spouse and the proxy consent process was approved by the Hamilton Integrated Research Ethics Board. Once the participant’s behavioural responsiveness had improved to a level at which consistent and accurate yes/no responses were possible, ongoing informed consent for further participation in the study was confirmed verbally directly from the participant. This was done by reading the consent statement to the participant and asking for a yes or no response. The verbal consent procedure was approved by the Hamilton Integrated Research Ethics Board.

**Figure 4:**
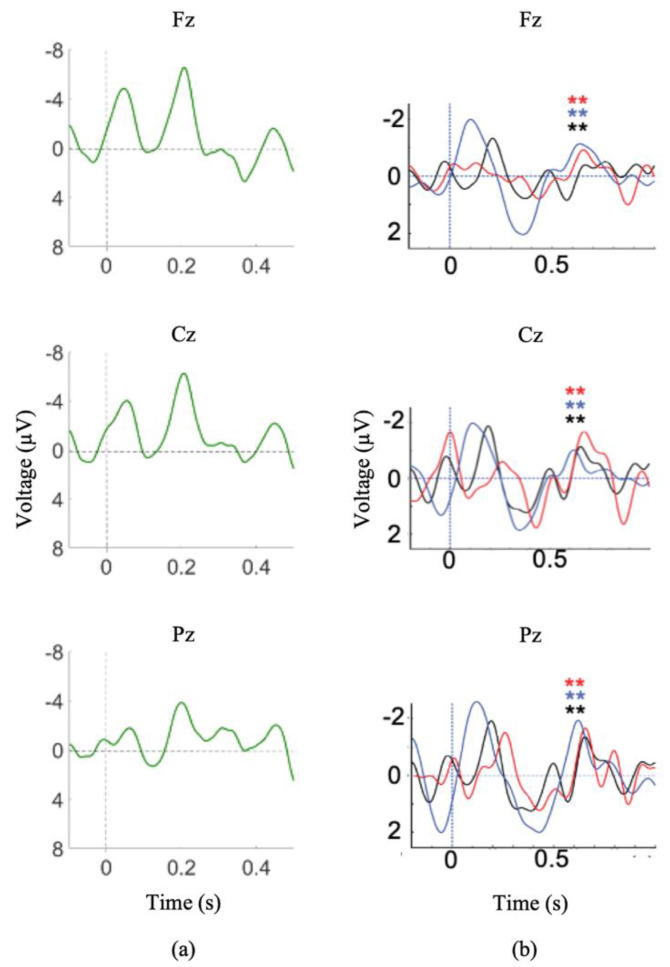
Module 3 ERP responses I. (**a**) Averaged waveforms for common first names stimuli, N1 and P2 waveforms are present. (**b**) Difference waves for incongruent/congruent stimuli (SVS paradigm) during Session 1(black), Session 2 (red), and Session 4 (blue). ** indicates significant difference between congruent and incongruent waveforms (*p* < 0.05). Note: a 7 Hz smoothing filter was applied for visualization only.

At the time of testing session 1 (2 months post-injury) the patient was no longer on sedatives, exhibited spontaneous eye opening, flexion response to pain, and a score of 8 on the Glasgow Coma Scale (GCS). A variation of the Subject’s Own Name P300 Paradigm was administered (stimuli included 7 common first names and the participant’s own name) and clear N1 and P2 ERPs were observed in the averaged waveforms corresponding to the common names (Figure 4a). The presence of these exogenous ERPs confirms that the patient was hearing the auditory stimuli (Figure 4a). Additionally, significant P300 responses were observed in response to the patient’s own name. 

During session 2 (5 months post-injury), the patient exhibited visual fixation and pursuit, as well as inconsistent yes/no responses (eye gaze or thumbs up). The SVS paradigm was administered during both testing sessions 1 and 2 and the patient demonstrated clear N400 responses to the paradigm both times (Figure 4b). 

**Figure 5:**
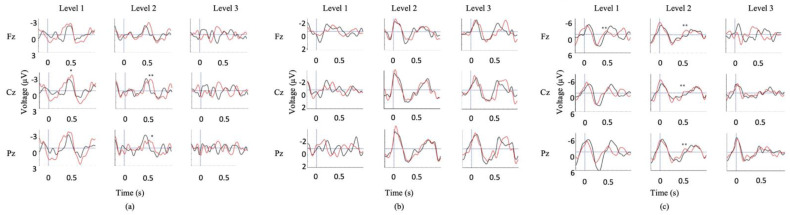
Module 3 ERP responses II. (**a**) Averaged N400 waveforms for auditory PPVT-R stimulus presentation (Session 3), (**b**) Averaged N400 waveforms for fast visual PPVT-R stimulus presentation (Session 4). (**c**) Averaged N400 waveforms for slow visual PPVT-R stimulus presentation (Session 5). Red waveforms are the averaged incongruent stimuli; black waveforms are the averaged congruent stimuli. Significant difference between congruent and incongruent waveforms (* *p* < 0.1, ** *p* < 0.05). Note: a 7 Hz smoothing filter was applied for visualization only.

Testing session 3 took place 6 months post-injury and the patient was able to use gestures to accurately answer yes/no questions with 80% accuracy. Due to suspicions of the participant fatiguing during previous SVS recordings, a paradigm of shorter duration was sought in order to capitalize on the available attention span. During session 3, an auditory version of the Peabody Picture Vocabulary Task (PPVT-R) was administered, which assessed receptive vocabulary by presenting visual images with either a matching or mismatching auditory word. The PPVT-R was divided into 3 levels of difficulty: level 1 consisted of preschool level (age 2.5–5 years-old) words, level 2 consisted of child-adolescent level (age 10–17 years-old) words, and level 3 consisted of adult level (advanced vocabulary) words. The picture was presented on the screen for a pre-set duration, then a pre-recorded word was played. This paradigm has previously been validated in healthy controls [66,67] as well as in participants with motor and communication impairments [68]. Significant N400 responses were recorded during the PPVT-R of session 3 (Figure 5a). 

By testing session 4 (7 months post-injury) the patient was consistently providing accurate yes/no responses and was beginning to vocalize. During session 4, the SVS paradigm was re-administered and significant N400 responses were recorded (Figure 4b). In order to further reduce the length of the paradigm (to continue to cope with patient fatigue), a visual version of the computer adapted PPVT-R was presented. Clinically, this paradigm may also be used to probe whether or not the participant is able to attend to text. The computer-adapted visual PPVT-R assessed receptive vocabulary by presenting visual pictures with matching or mismatching printed visual words. The visual PPVT-R was divided into the same 3 levels of vocabulary as the auditory PPVT-R. The picture was presented on the screen for a pre-set duration, then a printed word appeared on the screen above it. The PPVT-R of testing session 4 presented the printed word on the screen for a total of 750 ms. Following the session, the patient reported that he was unable to read the visual word due to the speed of presentation. 

The computer adapted visual PPVT-R was again administered in Session 5 (7.5 months post-injury) with the duration of presentation of the printed word increased to 1250 ms. After this session, the patient reported that he was able to successfully read the words.

Upon discharge, the patient had made “remarkable gains in his ability to communicate” scoring a 27/113 on the Western Neuro Sensory Stimulation Profile (WNSSP) at admission and a 110/113 at discharge.

### 3.2. Module 3: Analysis

Using EMSE Suite Data Editor (Version 5.4) (San Diego, CA: Source: Signal Imaging Inc.), a 0.1–30 Hz bandpass filter was applied offline, data was referenced to the mastoids, and electrooculogram artifacts were removed using independent component analysis. Trials were segmented into 1200 ms epochs (200 ms pre-stimulus to 1000 ms post-stimulus presentation). Epochs containing deviations of ±100µV were removed. Trials from the congruent and incongruent conditions were averaged separately. N400s were identified by visual inspection in the averaged waveforms as negative peaks in the incongruent condition between 300–700 ms by an experienced electrophysiologist (J.F.C.). Statistical differences between congruent and incongruent conditions during the N400 time window were evaluated using EMSE’s permutation test, which is appropriate for individual datasets and does not assume normality.

### 3.3. Module 3: Results

Waveforms generated from the SVS paradigm in sessions 1, 2, and 4 showed significant differences between congruent and incongruent stimuli (Figure 4b). Waveforms generated from session 3 auditory PPVT-R testing showed significant differences between congruent and incongruent stimuli in level 2 (Fz and Cz) (Figure 5a). Waveforms generated from session 4 PPVT-R testing showed no significant differences between congruent and incongruent stimuli (Figure 5b). Waveforms generated from session 5 PPVT-R testing showed significant differences in Level 1 (Fz) and Level 2 (Fz, Cz, Pz) between congruent and incongruent stimuli, but no differences in Level 3 (Figure 5c). These results suggest that the longer visual stimulus presentation enabled the patient to process semantic differences until the level of vocabulary became too advanced, indicating intact language processing functions. The differences between sessions is not attributable to the patient’s recovery, as N400 waveforms were observed in the auditory version of the adapted PPVT-R, recorded one month previous to the visual versions (session 3), as well as consistently in a SVS paradigm (sessions 1, 2, and 4), recorded at 2, 5, and 7 months post-injury. These results provide a strong example of how long-latency ERPs can be attenuated by rapid stimuli presentation in a single DOC patient. 

## 4. Case Examples from Unresponsive Populations 

We present data from 3 case studies outlined below. Through the application of strong paradigms, incorporating appropriate stimulation speeds, as well as instructions for participants to pay attention to the relevant stimuli, we demonstrate that long-latency ERPs can be elicited at the single subject level in unresponsive individuals. Data collection for each case study was approved by the Hamilton Integrated Research Ethics Board who approved the studies in accordance with the ethical standards of the Declaration of Helsinki. For each case study involving a participant 18 years of age or older (Case Study 2 and Case Study 3), the ability to provide informed consent was assessed through behavioural testing that determined whether or not each participant could consistently follow commands or provide accurate yes/no responses. If the participant was deemed unable to provide informed consent, informed consent was obtained through a substitute decision maker. The proxy consent process for both Case Study 2 and Case Study 3 was approved by the Hamilton Integrated Research Ethics Board.

### 4.1. Case Study 1

Continuous EEG was recorded from three electrode sites (Fz,Cz,Pz) in a 14-year-old male. The participant was diagnosed with non-verbal autism, using the Autism Diagnostic Observation Schedule 1 (ADOS−1). The participant listened to computer adapted auditory PPVT-R as well as a SVS paradigm. Written informed consent was obtained from the participant’s parents prior to study commencement.

Data was pre-processed as described in Module 1: Analysis. Offline bandpass filtering (0.5–15 Hz) was applied to the data. Data were re-referenced to the average of the mastoids. N1/P2 waveforms were sought in response to the average of the congruent word stimuli. For the N400 analysis, two-tailed serial t-tests were applied on single trials across four overlapping windows of length 40 ms. Time points with *p* < 0.05 were considered significant.

**Figure 6:**
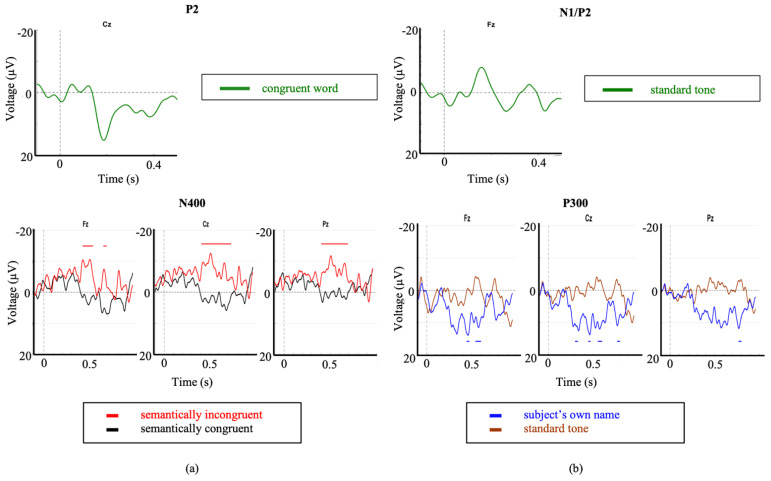
Event related potentials corresponding to Case Study 1 and Case Study 2. Exogenous ERPs were observed in response to: (**a**) the congruent word stimuli as part of the auditory PPVT-R paradigm (P2), and (**b**), the standard tone of the Modified Fischer paradigm (N1/P2). Significant differences were observed (significant intervals indicated by color-matched bars) for brain responses to (**a**) the SVS paradigm in the first case study, and (**b**) SON as part of the modified Fischer paradigm in the second case study.

A clear P200 was observed in response to average of the congruent word stimuli of the auditory PPVT-R, confirming that the participant was hearing the auditory paradigms. Furthermore, significant N400s to the SVS paradigm were elicited (Figure 6a). These responses may be used to assess this individual’s level of semantic comprehension.

### 4.2. Case Study 2

A 77-year-old (at the time of testing) female was involved in a motor vehicle collision resulting in severe traumatic brain injury causing a left hemispheric stroke, brain sheering, and a skull fracture. Computed tomography scans revealed acute epidural hematoma and acute subarachnoid hemorrhaging with no herniation or midline shift. The participant was diagnosed as minimally conscious state (MCS) 1 year following the motor vehicle collision. She was recruited for an ERP study 2 years after the date of injury and, at the time of testing, demonstrated no consistent means of communication. Continuous EEG was recorded from nine electrode sites as the participant listened to the Modified Fischer paradigm. Data were processed and analyzed following the methods described in the previous section (Case Study 1). Written informed consent for participation was obtained from the participant’s spouse prior to testing. 

Clear N1 and P2 responses were observed in response to the standard tone as part of the Modified Fischer paradigm confirming that the participant was hearing the auditory stimuli (Figure 6b). Significant and robust P300 responses were observed in response to the subject’s own name (SON) stimulus within the Modified Fischer paradigm (Figure 6b). These responses are indicative of a retention of some degree of conscious processing which is consistent with their MCS diagnosis.

### 4.3. Case Study 3

A 29-year-old male involved in a motor vehicle collision was recruited for an ERP study 58 days post-trauma. Tests of wakefulness and responsiveness were consistent with a diagnosis of unresponsive wakefulness syndrome (UWS; Glasgow Coma Scale = 4; Coma Recovery Scale-Revised = 4). Computed tomography scans conducted 43 days post-trauma revealed diffuse axonal injury, acute traumatic subarachnoid hemorrhage and a right parietal subdural hematoma. Additionally, signs of increased intracranial pressure were observed. A baseline EEG was conducted 21 days post-injury and the report stated: ‘‘This is an abnormal video EEG due to the presence of generalized slowing over the background activity. The above-described frontally dominant alpha-like activity may suggest alpha coma in evolution. Poor prognostic features from the current EEG include the paucity of waveforms as well as the lack of response to multiple afferent stimuli. These findings are in keeping with the clinical diagnosis of diffuse axonal injury.’’ A substitute decision maker provided written informed consent for participation in the study prior to testing. 64-channel EEG was recorded as the participant listened to the Modified Fischer Paradigm. 

EEG activity from 8 artifact-free electrodes was segmented into windows of 100 ms prior to and 1000 ms after auditory stimulus presentation. Windows were averaged within the standard tone, deviant tone, own name, and unfamiliar novel sound stimulus types. For each stimulus type, waveforms were compared point-by-point using serial t-scores to identify significant differences over time [45]. N1s and P2s were sought in response to the average waveforms corresponding to the standard tone. P300s were sought in SON-standard tone and the novel sound-standard tone subtraction waves. Waveform comparisons were conducted using the serial t-score method with differences significant at *p* < 0.05.

**Figure 7:**
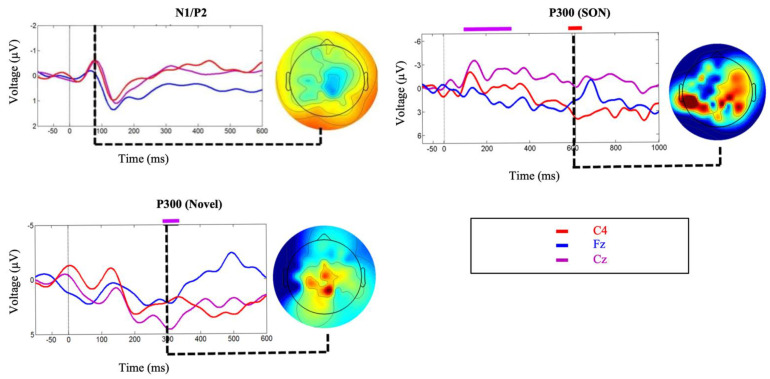
Event related potentials corresponding to Case Study 3. Exogenous ERPs were observed (overlaid on participant averages) in response to the standard tone of the Modified Fischer paradigm (N1/P2). Significant differences were observed (overlaid on participant averages) for brain responses to novel stimuli and SON as part of the Modified Fischer paradigm. Topographic maps at each point of significance show dominant activity in the posterior areas of the brain, decentralized, as typical of many brain-injured individuals. Figure 7 has been adapted from Blain-Moraes et al. 2016, “Figure 2” [69] and is licensed under the CC BY 4.0 (https://creativecommons.org/licenses/by/4.0/. Accessed on: 27 April 2017).

Clear N1 and P2 responses were elicited by the standard tone stimulus (Figure 7) confirming that the participant was hearing the auditory stimuli. Significant and robust P300 brain responses were elicited by the Modified Fischer Paradigm in response to the subject’s own name (SON) and novel stimulus (dog bark) (Figure 7). These responses are indicative of a retention of some degree of conscious processing. 1-month following the study, the patient had clinically recovered consciousness (Glasgow Coma Scale = 14; Coma Recovery Scale-Revised = 23) and was capable of responding to commands as well as verbally respond in conversation. The authors note that data from this participant has previously been published in Blain-Moraes et al. 2016 [69]. 

## 5. Discussion 

EEG-based measures are particularly useful in the assessment of behaviourally unresponsive individuals. The first objective in testing unresponsive patients is to establish objective evidence of sensory, perceptual and cognitive function. ERPs are well placed to accomplish this task, with over a half century of research relating specific ERP features to specific functions. Although ERPs are unable to provide neuroanatomical information, this is secondary to the assessment of whether the patient exhibits responses reflecting speech comprehension, automatic and intentional attentional processes, and a range of other important functions that other research has repeatedly demonstrated has diagnostic and prognostic implications [35,61,70]. Another advantage of EEG/ERPs is that it is relatively rare that a patient cannot be tested; a case that cannot be made for valuable but less flexible measures such as MRI and its multitude of related measures including fMRI, DTI and SWI, where upwards of 50% of relevant patients present with conditions that are incompatible with the technology. However, while ERPs present enormous advantages for assessing unresponsive individuals, the very ease of employing this tool can lead to a false sense of security in the presumed ease of designing robust studies and generating valid interpretations of the data. When working with unresponsive populations, paradigm design becomes even more critical as feedback cannot likely be provided by the patients. Thus, this paper reflects on several issues that can significantly affect the success of an ERP study of consciousness and presents a series of factors to consider when using this technology to assess levels of consciousness in unresponsive individuals. 

First, we have presented evidence supporting the importance of choice of paradigm when attempting to elicit long-latency ERPs. It is essential to ensure a paradigm’s appropriateness for the needs of the proposed research before testing any patient population. Between the Modified Fischer and the Names paradigm, both designed to elicit P300s, the Modified Fischer produced significantly larger responses. Similarly, between the SVS and WWP paradigms, both designed to elicit N400s, the SVS paradigm generated significantly more robust responses in healthy controls. These discrepancies are unsurprising. There is ample evidence that the N400 component is fundamentally related to the strength of the expectation produced by the context in which a word is found, and that the context provided by discourse results in a larger N400 than a sentence [71], which in turn is larger than word-word priming [72]. Thus, the most effective experimental manipulations should be used when testing for consciousness in unresponsive individuals. Minimally, this involves ensuring that the paradigm elicits the desired response in healthy conscious controls before using it to assess the state of consciousness of unresponsive individuals. Our results support the use of strong context paradigms to elicit N400s within the limitations presented by patient stamina and fatigue. It is clear from our healthy control data that word-word priming is not the most effective paradigm to be used when the investigation involves the analysis of individual subjects/patients (Figure 1d and Figure 2d), but that incongruent words embedded in sentence contexts result in clear N400 responses (Figure 1c and Figure 2c). The successful use of this paradigm to elicit ERPs in a patient diagnosed with unresponsive wakefulness syndrome (UWS) is illustrated in Figure 5b, and has been used as evidence to reflect the presence of speech comprehension in unresponsive individuals [9]. Further evidence of the strength of the Modified Fischer paradigm can be seen with Case Study 3 (Figure 7) in which a patient diagnosed with UWS exhibited a P300 in response to his own name.

Second, we provide support for the factor of age, which must be considered carefully insofar as the etiologies of unresponsiveness (e.g., stroke, traumatic brain injury) often involve individuals of markedly different ages. We demonstrate that exposure to 4 different paradigms showed notable age differences (Figure 1 and Figure 2), where the older cohort demonstrates significantly attenuated amplitudes, particularly in the N400 SVS Paradigm. These data reaffirm the notion that the interpretation of the amplitude of the elicited ERP must be treated with caution, and that the participant’s age significantly modifies the expected ERP characteristics. Thus, it is important that studies explicitly account for the age of the participant when interpreting ERP data. 

Next, we reiterate that stimulation rate needs to be monitored carefully when testing unresponsive individuals. It is has been well-established that the N400 is exquisitely sensitive to stimulus presentation characteristics, including accelerated word presentation [72], and stimulus degradation [73,74,75]. As illustrated in Figure 5, absence of response may reflect an attenuated ability to perceive the stimuli, rather than a lack of consciousness in unresponsive individuals. Experimenters may consider re-testing at a slower stimulus presentation speed those individuals who show no ERP responses initially, to ensure that every opportunity is explored before making judgements about an individual’s state of consciousness. 

We demonstrate that by employing strong paradigms with stimuli presented at appropriate speeds, factoring individual age into the interpretation, we are able to successfully elicit long-latency ERPs from unresponsive populations both at the individual and small sample size levels. Within the included case studies, an individual with severe autism demonstrates significant N400s to the SVS paradigm, which can then be used to assess his level of receptive vocabulary (Figure 6a). Two individuals diagnosed with UWS demonstrate robust P300 responses in response to the Modified Fischer paradigm (Figure 6b,c), indicating that they retain some degree of conscious processing. 

### Limitations

In this paper, we discuss three critical factors that need to be accounted for when eliciting ERPs in behaviourally unresponsive individuals. There are several limitations to the analyses supporting our recommendations. First, the data presented in modules 1 and 2 are generated from small samples sizes (i.e., n = 5 per group). While this appears to be small for supporting our recommendations, we believe that they provide sufficient evidence to support the effects of stimulation paradigm and age on ERP elicitation for several reasons: our data are a randomly selected subset of participants taken from a larger sample (i.e., n = 26, n = 13) from Mah & Connolly 2018 [60], and show results consistent with those generated in the larger population, and; in order to have clinical utility, the process of eliciting and analyzing ERPs must be effective on a single-subject basis. Factors that have effects on the group-level, but do not translate to all individual subjects cannot be manipulated to reliably produce ERPs in an individual unresponsive participant. The ability to see the mitigating effects of the factors presented in modules 1 and 2 in our small sample supports the importance of accounting for these factors when working with this population. Finally, while strong conclusions can be drawn based on the presence of an ERP (i.e., positive results), no claims regarding the level of consciousness of an individual can be made in the absence of an ERP (i.e., negative results). In other words, the inability to detect a long-latency ERP is not sufficient to diagnose an absence of consciousness. 

## 6. Conclusions

Much of the current debate regarding the relationship between long-latency ERPs and states of consciousness has resulted from data collected from studies that do not adequately consider each of these three critical factors (e.g., [41,47]). The data presented in this paper provide small sample and individual level evidence that several factors influence both the presence and the morphology of the P300 and N400 waveforms, and underscore the exquisite care that researchers must undertake in the design of ERP studies. While long-latency ERPs have the potential to increase the accuracy of assessing levels of consciousness in unresponsive individuals, interpretation of data collected from this population will ultimately be ambiguous unless the factors presented here are carefully controlled. We encourage the application of our recommended practices in all ERP studies of consciousness, enabling more accurate and reliable diagnosis and assessment of individuals who are behaviourally unresponsive. 

## Data Availability

All data files related to this manuscript are available from the Scholars Portal Dataverse (https://doi.org/10.5683/SP/C7JAGN, accessed on: 6 July 2018). Data corresponding to Modules 1 and 2 can be found at doi:10.5683/SP/MZHKJT.
